# Differential Effects of Toll-Like Receptor Signaling on the Activation of Immune Responses in the Upper Respiratory Tract

**DOI:** 10.1128/spectrum.01144-21

**Published:** 2022-02-23

**Authors:** Meiyi Xu, Ning Li, Xin Fan, Ya Zhou, Shuai Bi, Adong Shen, Beinan Wang

**Affiliations:** a Key Laboratory of Pathogenic Microbiology and Immunology, Institute of Microbiology, Chinese Academy of Sciences, Beijing, China; b Savaid Medical School, University of Chinese Academy of Sciences, Beijing, China; c Beijing Pediatric Research Institute, Beijing Children's Hospital, National Center for Children's Health, Beijing, China; UC Davis

**Keywords:** toll-like receptors, mucosal tolerance, respiratory inflammation, respiratory adaptive immune responses

## Abstract

Vaccination through the upper respiratory tract (URT) is highly effective for the prevention of respiratory infectious diseases. Toll-like receptor (TLR)-based adjuvants are immunostimulatory and considered potential adjuvant candidates. However, the patterns of immune response to different TLRs at the URT have not been revealed. In this study, SPF mice were preexposed to TLR agonists intranasally to simulate the status of humans. Inflammatory response to TLR agonists and TLR signal-mediated adaptive immune responses were analyzed. The results revealed that similar to human tonsils, inflammatory response to stimulation with TLR4 or TLR2 agonist was attenuated in agonist-exposed mice but not in mice without this exposure. In contrast, TLR9 or TLR3 agonist preexposure did not affect the inflammatory response to restimulation by matching agonists. For the adaptive immune response, after agonist preexposure the antibody response to antigens adjuvanted with TLR4 or TLR2 agonist was substantially restricted, whereas, both antibody and T cell responses to antigens adjuvanted with TLR9 or TLR3 agonist were activated as robustly as in mice without agonist exposure. Moreover, we demonstrate that the mechanisms underlying the differential activation of TLRs are regulated at the level of TLR expression in innate and adaptive immune cells. These results indicate that TLRs on the cell surface (TLR4 and 2) and in the endolysosomal compartments (TLR9 and 3) display distinct immune response patterns. The findings provide important information for the use of TLR agonists as mucosal adjuvants and enhance our understanding of immune responses to bacterial and viral infections in the respiratory mucosa.

**IMPORTANCE** Agonists of TLRs are potential adjuvant candidates for mucosal vaccination. We demonstrated that the TLR-mediated inflammatory and antibody responses in the URT of SPF mice exposed to extracellular TLR agonists were substantially restricted. In contrast, inflammatory and adaptive immune responses, including B and T cell activation, were not desensitized in mice exposed to intracellular TLR agonists. The distinct responsive patterns of extra and intracellular TLRs regulated at TLR expression in immune cells. The results indicated that TLRs differentially impact the innate and adaptive immune response in the URT, which contributes to the selection of TLR-based mucosal adjuvants and helps understand the difference between the immune response in bacterial and viral infections.

## INTRODUCTION

The upper respiratory tract (URT) mucosa is continuously exposed to the external environment and is an important site of immune regulation (1, 2). Vaccination through the URT has beneficial effects by eliciting immune defense, both locally and systemically (3), and is a highly effective and recommended method to prevent mucosally transmitted infections. The subunit vaccines are described as new-generation vaccines owing to their improved safety, such as excluding the risk of postvaccination infection and minimizing side effects associated with the use of whole bacterial cells (4). However, the poor immunogenicity of subunit vaccines due to the lack of immunostimulatory activity is a limitation for which adjuvants are required (5). For mucosal vaccines, most are live attenuated vaccines that do not require adjuvants, such as the intranasal vaccine for influenza virus (FluMist) and oral vaccine for rotavirus (Rotarix) (6). However, no subunit vaccine delivery through the mucosa is licensed for humans, which is mainly due to the lack of safe and effective mucosal adjuvants (7).

Toll-like receptors (TLRs) are a group of pattern-recognition receptors (PRRs). The activation of TLRs triggers innate immunity and is critical for the induction of the adaptive immune response, offering new potential for adjuvant discovery (8). TLRs are expressed on the cell surface (TLR-1, 2, 4, 5, 6, and 10) or inside cells (TLR-3, 7, 8, and 9). They recognize a diverse range of pathogen-associated molecular patterns (PAMPs) presented in many microbes, such as lipoteichoic acid from Gram-positive bacteria (TLR2), lipopolysaccharide (LPS) from Gram-negative bacteria (TLR4), viral dsRNA (TLR3), and unmethylated CpG oligodeoxynucleotide (TLR9) in virus and bacterial DNA (9, 10). TLRs in the URT play a critical role in protective immunity against pathogens while avoiding aberrant inflammatory response to the large numbers of commensals present in the URT mucosal surface (11).

The respiratory mucosal immune system shares common features with the intestinal system and displays distinctive immunological phenotypes (12, 13). Human tonsils are considered the nasal-associated lymphoid tissue (NALT) in URT mucosa and act as the first line of defense against inhaled microbes (14). Murine NALT is functionally a human tonsil homolog (15) capable of processing and presenting antigens for the initiation of antigen-specific immune responses (13, 16), and has been used as a surrogate to study URT mucosal immunity.

Laboratory mice living in “specific-pathogen-free” (SPF) barrier facilities have immunological features that are more in common with those of neonatal humans compared with pet store mice with diverse microbial experience (17). The immunological phenotypes of adult humans are simulated in laboratory mice that are frequently exposed to free-living conditions (17, 18). These findings indicate that TLR responses in the URT of SPF mice are different from those in adult humans, which may affect the translation findings in mice to humans where TLR-based adjuvants are concerned.

Most of the studies on TLR tolerance focus on systemic immune responses by administration of TLR ligands through intraperitoneal or intravenous injection (19–21). Although mucosal responses to TLR ligands have been studied for adaptive immune response, all of them are performed on SPF mice, which did not reflect the conditions in human adults. It is not known in a condition similar to that of humans which TLR agonists can induce tolerance in URT mucosa, and how different TLR signaling influences the adaptive immune response. In this study, URT mucosal responses to several TLR agonists were examined in NALTs of SPF mice and TLR agonist-experienced mice. Our data demonstrate that responses of extracellular TLRs to their agonists were desensitized in agonist-experienced mice but not in SPF mice. In contrast, the response of intracellular TLRs remained sensitive in agonist-experienced mice as well as in SPF mice. In addition, the distinct response patterns of extra- and intracellular TLRs differentially modulated the adaptive immune responses.

## RESULTS

### LPS exposure to NALT of SPF mice led to an attenuated inflammatory response similar to that in human tonsils.

**FIG 1:**
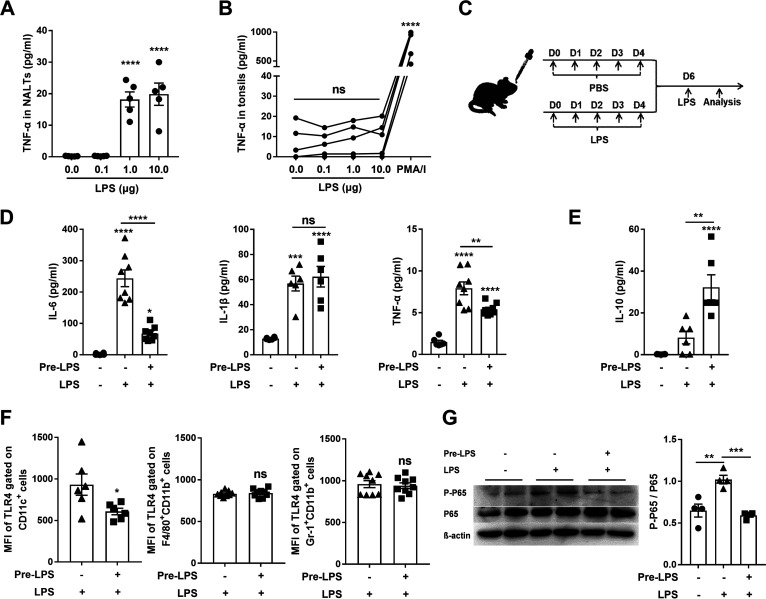
LPS exposure to NALT of SPF mice led to an attenuated inflammatory response similar to that in human tonsils. (A–B) The levels of TNF-α in the culture supernatant of NALT and tonsil cells were assessed by ELISA 24 h after LPS stimulation. (C) Experimental setup illustrating the timeline. (D–E) IL-6, IL-1β, TNF-α, and IL-10 production were measured in NALT supernatant by ELISA 2 h (D) or 12 h (E) after the LPS stimulation. (F) The mean fluorescence intensity (MFI) of TLR4 in NALTs was determined by FACS 2 h gated on CD11c^+^ monocytes or 6 h gated on macrophages (CD11b^+^F4/80^+^) and neutrophils (CD11b^+^Gr-1^+^) after the stimulation. Very few of CD11c^+^ monocytes, macrophages, and neutrophils were found in nontreated control mice. (G) The levels of phospho-NF-κB p65 in NALTs were measured by Western blot 30 min after the stimulation. (A, D, E, F, and G) are from 2–3 independent experiments (*n* = 4–9); (B) The dots connected by each line represent one donor (*n* = 5). Data are presented as means ± SEM; *, *P < *0.05, **, *P < *0.01, ***, *P < *0.001, ****, *P < *0.0001, ns, not significant. The asterisk above the bar indicates a significant difference compared to the first group.

LPS, an agonist of TLR4, induces inflammation on entering circulation, leading to microcirculatory dysfunction, tissue damage, and septic shock; in contrast, LPS inhalation mediates tolerance (22), suggesting that inflammatory response to LPS is affected by exposure to the external environment. We hypothesized that the response to LPS in the URT mucosa of SPF mice is different from human tonsils because SPF mice are less exposed to environmental microbes. NALT cells from SPF mice were exposed to various concentrations of LPS and culture supernatant was analyzed by ELISA. We found that TNF-α production was highly induced when LPS concentration reached 1 μg/mL and maintained at the high level when LPS concentration was 10-fold higher (10 μg/mL) (Fig. 1A). However, no increased TNF-α production was found in human tonsil samples in the presence of LPS at these concentrations compared with that in nontreated control cells, although TNF-α was highly induced in response to PMA/ionomycin (a positive control) (Fig. 1B). These results indicate that the response of URT mucosa to LPS in SPF mice is different from that in humans, which may play a role in the consistency of results between laboratory mice and humans.

Failure to induce an inflammatory response to LPS in the tonsils could result from constant exposure of the tonsils to environmental LPS-bearing bacteria. LPS content in the tonsil samples was examined. LPS was detected in all examined human tonsil samples (2.5–5.5 ng/mL/10^6^ cells) but was undetectable in NALT of SPF mice (Table 1), suggesting that the lower environmental exposure of SPF mice is responsible for the sensitivity to LPS. To simulate the environmental exposure of tonsils, SPF mice were pretreated with LPS intranasally for five consecutive days and stimulated with LPS intranasally 2 days later. NALT was harvested 2 h after stimulation (Fig. 1C). ELISA revealed that IL-6 and TNF-α production was robustly induced in cultured NALT cells from SPF mice but reduced in LPS-pretreated mice, although IL-1β was equally induced in both groups (Fig. 1D and S1A). Conversely, IL-10, an anti-inflammatory cytokine, was produced at higher levels in LPS-pretreated mice (Fig. 1E and S1B), suggesting that it might be involved in the reduced IL-6 and TNF-α. It has been reported that lower pro-inflammatory cytokine response to LPS in human tonsils is associated with the reduction of TLR4 expression in mononuclear cells (23). We found that TLR4 expression on cell surface examined by flow cytometry was decreased in NALT CD11c^+^ monocytes of LPS-pretreated mice compared with that in naive mice, while the expression in NALT macrophages (CD11b^+^F4/80^+^) and neutrophils (CD11b^+^Gr-1^+^) was comparable in both groups (Fig. 1F and S2A). Because TLR4 activation induces inflammatory cytokines through the NF-κB pathway Western blots were performed on NF-κB activation. The phosphorylation of NF-κB P65 in NALT was diminished in LPS-pretreated mice (Fig. 1G), suggesting that constant LPS exposure desensitizes mucosal inflammatory response to LPS through reduction of TLR4 expression in CD11c^+^ monocytes and NF-κB activation.

**TABLE 1:** LPS was detected in human tonsil samples but not in NALTs of SPF mice

	LPS^*a*^/supernatant (ng/mL/10^6^ cells)	
No.	NALTs of SPF mice	Human tonsils
1	0	3.3
2	0	5.5
3	0	3.2
4	0	3.8
5	0	2.5
6	0	4.5

a LPS quantitation of NALT and tonsil supernatant was tested using Chromogenic Tachypleus Amebocyte Lysate (TAL) test kit (sensitivity > 0.125 ng/mL) (*n* = 6).

### Exposure of respiratory mucosa to LPS reduced B cell but not T cell responses.

**FIG 2:**
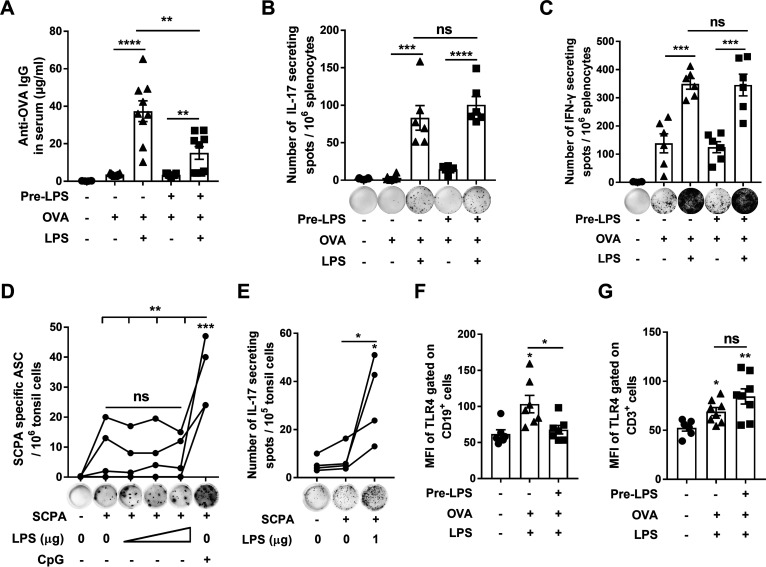
Exposure of respiratory mucosa to LPS reduced B cell but not T cell responses. Mice were pretreated with LPS intranasally for five consecutive days and then immunized with OVA or OVA/LPS 2 days later. (A) Serum antibodies were measured by ELISA; (B–C) OVA-specific IL-17^+^ and IFN-γ^+^ cells were determined by ELISpot assays 14 days after immunization. (D–E) SCPA-specific IgA^+^ antibody-secreting cells and IL-17^+^ cells in tonsil samples were determined by ELISpot assays. (F–G) The MFI of TLR4 gated on CD19^+^ and CD3^+^ cells in NALTs was measured by FACS 6 h after immunization. (A, B, C, F, and G) are from 2–3 independent experiments (*n* = 6–9). (D–E) The dots connected by each line represent one donor (*n* = 4). All the data are presented as means ± SEM; *, *P < *0.05, **, *P < *0.01, ***, *P < *0.001, ****, *P < *0.0001, ns, not significant. The asterisk above the bar indicates a significant difference compared to the first group.

To determine the impact of LPS on adaptive immune responses, SPF mice were pretreated with LPS as before and immunized intranasally with ovalbumin (OVA) alone or OVA with LPS 2 days later when the cytokine responses returned to the baseline. OVA-specific serum IgG and splenic T cell responses were measured 14 days after immunization. IgG was substantially induced in response to OVA/LPS but not to OVA alone in the mice without LPS pretreatment, indicating that LPS has adjuvant activity in SPF mice. However, in LPS-pretreated mice, antibody production was considerably lower (Fig. 2A). In contrast, T cell responses examined by ELISpot revealed that IL-17 or IFN-γ secreting T cells were strongly increased in response to OVA/LPS regardless of LPS pretreatment (Fig. 2B and C). We next investigated B and T cell responses in human tonsils. A streptococcal antigen, C5a peptidase (SCPA), was used to examine the antigen-specific B cell response in tonsils (24) given that Streptococcus pyogenes is a pathogen frequently found in tonsillitis (25). ELISpot assays showed that IgA-secreting cells were not significantly induced following stimulation with SCPA alone relative to unstimulated tonsil cells. No further increases in the numbers of these cells were observed following stimulation with SCPA/LPS when even higher doses of LPS were used (Fig. 2D). However, substantially higher levels of these cells were found following stimulation with SCPA and CpG (positive control). For tonsil T cell responses, SCPA-specific IL-17-secreting cells were significantly higher in the cells stimulated with SCPA/LPS than those with SCPA alone (Fig. 2E). These results indicate that LPS exposure attenuates LPS-promoted activation of B cells but not T cells.

TLRs of B and T cells also play an important role in adaptive immune responses (26, 27). Flow cytometry assays showed that after stimulation with OVA/LPS, TLR4 expression on cell surface was increased in NALT B cells in SPF mice but not in SPF mice pretreated with LPS (Fig. 2F and S2B); whereas, TLR4 expression was increased in NALT T cells of mice regardless of LPS pretreatment (Fig. 2G and S2B). These results indicate that the differential responses of B and T cells to LPS exposure are related to the regulation of TLR4 expression on these cells and suggests that B cell activation requires TLR4 signaling from CD11c^+^ monocytes and B cells, whereas T cell activation relies more on its TLR4, which is intolerant to LPS.

### CpG exposure did not attenuate the inflammatory response and CpG-adjuvanted adaptive immune responses in NALT.

**FIG 3:**
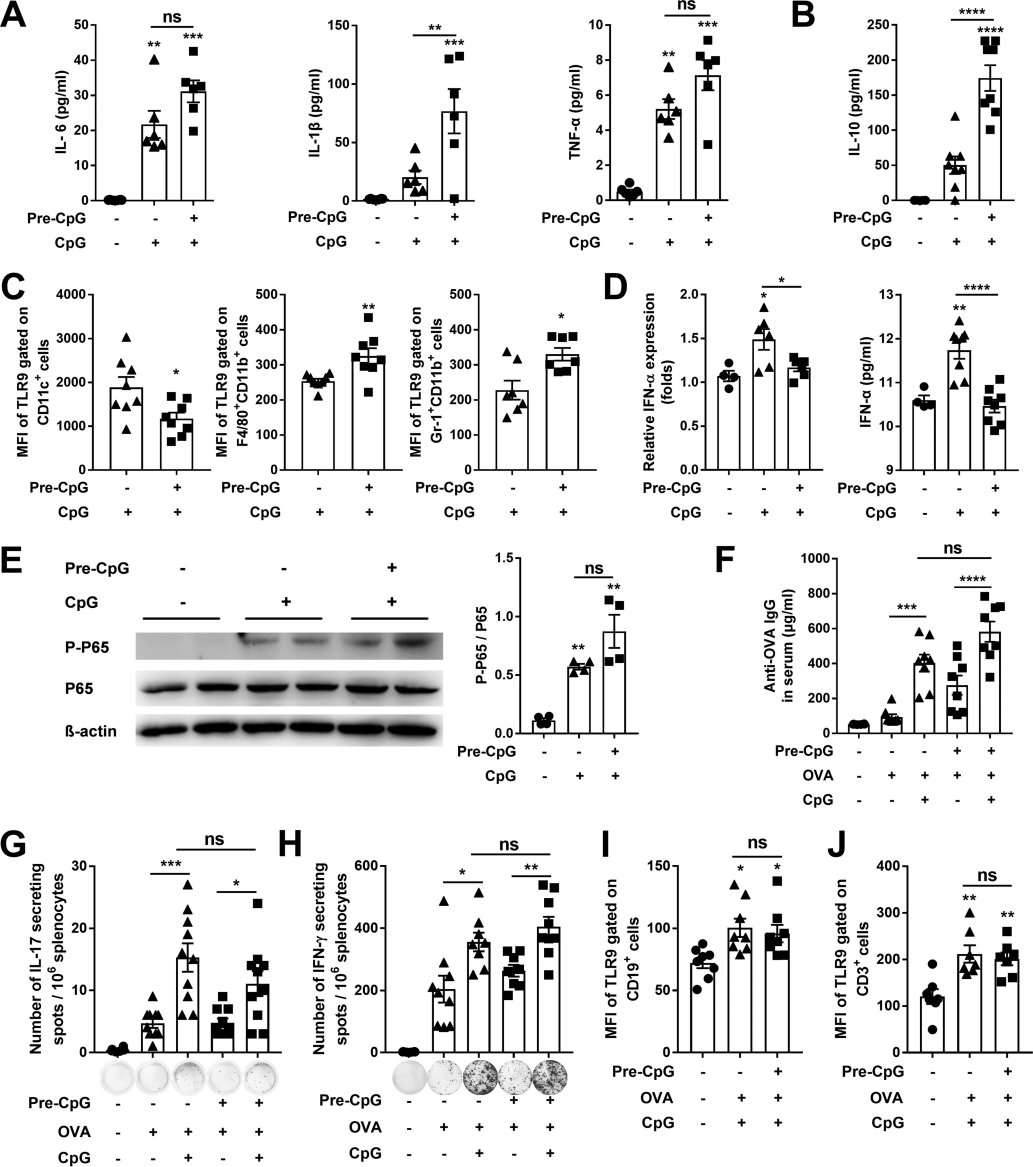
CpG exposure did not attenuate the inflammatory response and CpG-adjuvanted adaptive immune responses in NALT. The experimental timeline is described in (Fig. 1C). (A–B) The production of IL-6, IL-1β, TNF-α, and IL-10 was measured in NALT supernatant by ELISA 2 h (A) or 12 h (B) after the CpG stimulation. (C) The MFI of TLR9 in NALTs was determined by FACS 2 h (CD11c^+^ monocytes) or 12 h (macrophages and neutrophils) after the stimulation. Very few of CD11c^+^ monocytes, macrophages, and neutrophils were found in nontreated control mice. (D) The relative IFN-α expression and the IFN-α production of NALTs were measured by qRT-PCR and ELISA 6 h and 12 h after the stimulation, respectively. (E) The levels of phospho-NF-κB p65 in NALTs were measured by Western blot 90 min after the CpG stimulation. The experimental timeline is described in (Fig. 2). (F) Serum antibodies were detected by ELISA; (G–H) OVA-specific IL-17^+^ and IFN-γ^+^ cells in splenocytes were determined by ELISpot assays. (I–J) The MFI of TLR9 gated on CD19^+^ and CD3^+^ cells of NALTs was determined by FACS 2 h after immunization. Data are the mean ± SEM of 2–3 independent determinants (4–10); *, *P < *0.05, **, *P < *0.01, ***, *P < *0.001, ****, *P < *0.0001, ns, not significant. The asterisk above the bar indicates a significant difference compared to the first group.

CpG is an effective mucosal adjuvant in humans (28, 29), suggesting that mucosal immune response to CpG is different from LPS. However, the underlying mechanisms are not clear. We showed that tonsillar B cells responded to SCPA/CpG (Fig. 2D), indicating that CpG does not induce tolerance. To test this possibility, mice were pretreated with CpG in the same way as for pretreatment with LPS. Cytokines in NALT determined by ELISAs revealed that unlike the responses to LPS, the levels of IL-6, IL-1β, and TNF-α were increased in response to CpG stimulation in CpG-pretreated mice, with levels comparable to or even higher than those in mice without CpG pretreatment (Fig. 3A). In the cultured human tonsil cells, the TNF-α response to CpG showed a trend of dose-dependent manner as measured by ELISA without statistical significance (Fig. S3). Similar to the response to LPS in LPS-pretreated mice, IL-10 was increased in CpG-pretreated mice (Fig. 3B). Flow cytometry analysis of NALT cells revealed that TLR9 expression was reduced in CD11c^+^ monocytes but increased in macrophages and neutrophils of CpG-pretreated mice (Fig. 3C). IFN-α response to CpG in NALT was examined to corroborate the TLR9 reduction in CD11c^+^ monocytes because IFN-α is a primary response of plasmacytoid dendritic cells (pDCs) to CpG and repeated treatment of pDCs with CpG reduced IFN-α production (30, 31). Consistent with these reports, RT-PCR, and ELISA showed that IFN-α was increased following CpG stimulation in mice without CpG pretreatment but not in mice pretreated with CpG (Fig. 3D), indicating that IFN-α response to CpG restimulation is tolerant. Since TLR9 recognizes viruses and intracellular bacteria and induces IFN-α to inhibit viral and bacterial replication, the reduction of TLR9 in CD11c^+^ monocytes suggests that it may represent negative feedback of IFN-α, possibly through induction of IL-10. Western blot revealed that phosphorylation of NF-κB p65 in NALT was induced in response to CpG, regardless of CpG pretreatment (Fig. 3E). These results suggest that CpG exposure reduces TLR9 expression in CD11c^+^ monocytes, leading to attenuation of IFN-α production; but not in macrophages and neutrophils, resulting in an unaffected inflammatory response to CpG restimulation through activation of NF-κB.

To verify a link between the adjuvant effectiveness and the intolerant response to CpG, mice were pretreated with or without CpG as before and immunized with OVA/CpG intranasally. Antibody and T cell responses were determined 14 days after immunization. Unlike in LPS-pretreated mice, the levels of serum IgG were elevated following OVA/CpG stimulation with similar levels regardless of CpG pretreatment (Fig. 3F). The numbers of splenic IL-17 and IFN-γ-secreting cells (Fig. 3G and H), as well as TLR9 expression in B and T cells (Fig. 3I and J), were also increased in the two groups of mice without a significant difference. These data indicate that CpG exposure does not affect CpG-promoted activation of B and T cell responses, which may explain the mucosal adjuvant activity of CpG in humans.

### Effects of TLR2 and TLR3 signaling on mucosal immune responses were similar to those of TLR4 and TLR9, respectively.

**FIG 4:**
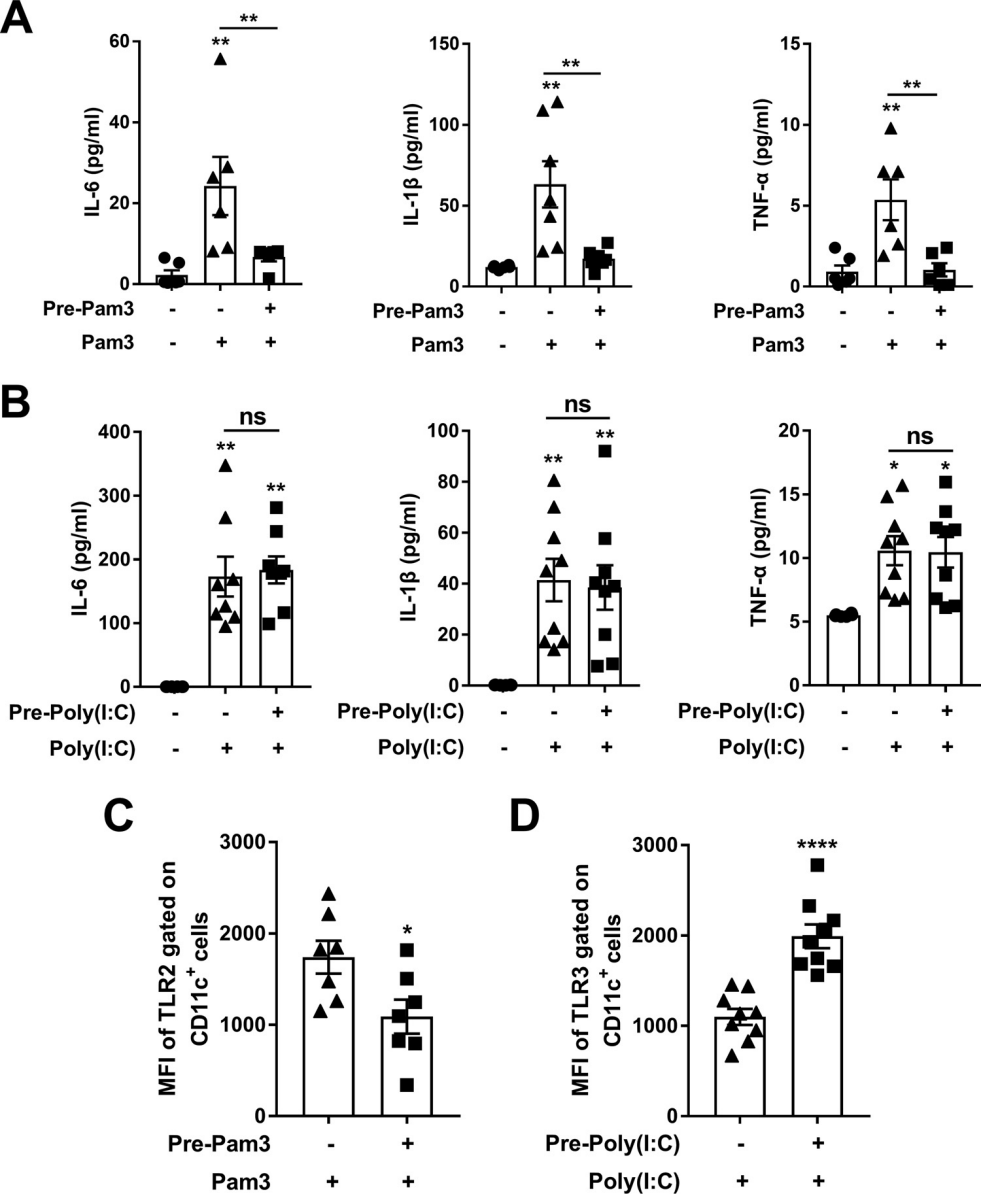
Effects of TLR2 and TLR3 signaling on mucosal immune responses were similar to those of TLR4 and TLR9, respectively. The experimental timeline is illustrated as in (Fig. 1C). (A–B) The production of IL-6, IL-1β, and TNF-α was measured in NALT supernatant by ELISA. (C–D) The MFI of TLR2 and TLR3 gated on CD11c^+^ monocytes in NALTs was determined by FACS 2 h after the stimulation. Very few of CD11c^+^ monocytes were found in nontreated control mice. Data are the mean ± SEM of 2–3 independent determinants (*n* = 6–9); *, *P < *0.05, **, *P < *0.01, ****, *P < *0.0001, ns, not significant. The asterisk above the bar indicates a significant difference compared to the first group.

**FIG 5:**
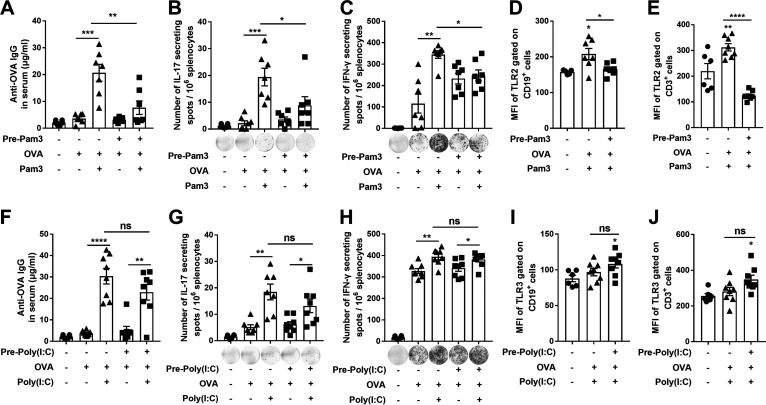
The adaptive immune response was desensitized by the extracellular TLR2 agonist but not by the intracellular TLR3 agonist. The experimental timeline is illustrated as in (Fig. 2). (A and F) Serum antibodies were determined by ELISA; (B, C, G and H) OVA-specific IL-17^+^ and IFN-γ^+^ cells in splenocytes were determined by ELISpot assays 14 days after immunization. (D, E, I, and J) The MFI of TLR2 and TLR3 gated on CD19^+^ and CD3^+^ cells in NALTs was determined by FACS 2 h after immunization. Data are the mean ± SEM of 2–3 independent determinants (*n* = 6–8); *, *P < *0.05, **, *P < *0.01, ***, *P < *0.001, ****, *P < *0.0001, ns, not significant. The asterisk above the bar indicates a significant difference compared to the first group.

The differential mucosal immune responses to LPS and CpG suggest that the extracellular and intracellular TLRs act differently. An extracellular TLR2/1 agonist, Pam3CSK4, and an intracellular TLR3 agonist Poly (I:C) (synthetic double-stranded RNA polyriboinosinic polyribocytidylic acid) (32) were tested. Mice were pretreated and stimulated with Pam3CSK4 or Poly (I: C) and cytokines in NALT were determined. IL-6, TNF-α, and IL-1β were substantially induced in mice without Pam3CSK4 pretreatment, but not in mice treated with Pam3CSK4 (Fig. 4A). Nevertheless, these cytokines were equally increased in response to Poly (I: C) stimulation regardless of Poly (I: C) pretreatment (Fig. 4B). Similar to TLR4 expression, TLR2 expression was reduced in Pam3CSK4-pretreated mice (Fig. 4C), whereas TLR3 was substantially enhanced in Poly (I: C)-pretreated mice (Fig. 4D). These results confirm that the inflammatory responses of extra- and intracellular TLRs to their agonists are characteristically different. The impact of TLR2 or TLR3 agonist exposure on adaptive immune responses was further determined. Agonist-pretreated mice were immunized with OVA as before. OVA-specific antibody production was substantially reduced in mice pretreated with Pam3CSK4 (Fig. 5A). Interestingly, different from LPS-pretreated mice in which T cell response was not attenuated, IL-17- or IFN-γ-producing T cells were also reduced in number in Pam3CSK4 pretreated mice compared with that in mice without the pretreatment (Fig. 5B and C). Consistent with these findings, TLR2 expression was increased in B and T cells of mice without Pam3CSK4 pretreatment but not in those of mice with the pretreatment (Fig. 5D and E). Conversely, no significant reduction of antibody or T cell responses was observed in mice pretreated with Poly (I: C) (Fig. 5F and G, and 5H), and no decrease in TLR3 expression in B and T cells were found in mice regardless of Poly (I: C) pretreatment (Fig. 5I and J). These results indicate that extracellular TLRs, such as TLR2 and 4, can be desensitized by exposure to their agonist, leading to attenuated inflammatory and antibody responses. In contrast, those of intracellular TLRs, such as TLR3 and 9, remain sensitive following exposure to their agonists, resulting in B and T cell activation that do not exhibit attenuation.

### The effects of TLR agonist exposure on immune responses to bacterial and viral infections.

**FIG 6:**
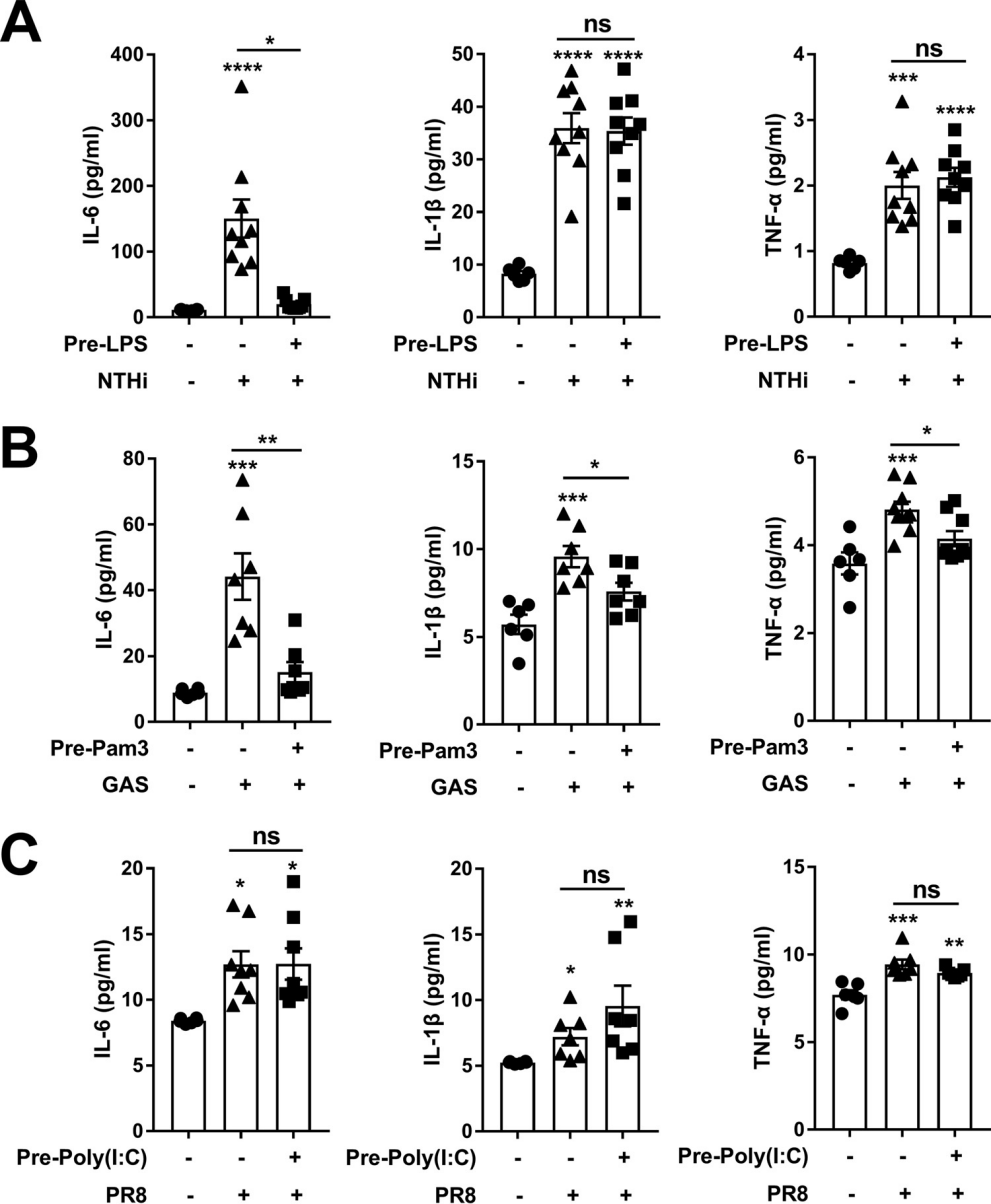
The effects of TLR agonist exposure on immune responses to bacterial and viral infections. Mice were pretreated with LPS, Pam3CSK4, or Poly (I: C) intranasally for five consecutive days, and then challenged with NTHi, GAS, or PR8 intranasally 2 days later, respectively. (A–C) The production of IL-6, IL-1β, and TNF-α in NALT supernatant was measured by ELISA at 6 h (A), 12 h (B), and 72 h (C) postinfection. Data are the mean ± SEM of 2–3 independent determinants (*n* = 6–9); *, *P < *0.05, **, *P < *0.01, ***, *P < *0.001, ****, *P < *0.0001, ns, not significant. The asterisk above the bar indicates a significant difference compared to the first group.

To determine the impact of TLR signaling on immune responses to respiratory bacterial infection, mice were pretreated with LPS and then challenged intranasally with nontypeable Haemophilus influenzae (NTHi), a Gram-negative bacterium bearing LPS. Cytokines in NALT were determined after challenge by ELISA. The results showed that IL-6 was robustly induced in mice without LPS pretreatment but not in LPS-pretreated mice, although IL-1β and TNF-α were induced equally in the two groups (Fig. 6A). Similar experiments were performed in mice with Pam3CSK4 and Streptococcus pyogenes (GAS), a Gram-positive bacterium containing Pam3CSK4. We found that the IL-6 response was also attenuated in Pam3CSK4-pretreated mice with decreased production of IL-1β and TNF-α following the challenge (Fig. 6B). Mice were also pretreated with Poly (I: C) and then challenged with influenza virus PR8. Unlike in the mice pretreated with LPS or Pam3CSK4, the production of IL-6, IL-1β, and TNF-α was increased in response to PR8 challenge to the same extent in the mice regardless of Poly (I: C) pretreatment (Fig. 6C). These results suggest that exposure to extracellular TLR agonist attenuates the inflammatory response to bacterial infection, whereas exposure to intracellular TLR agonists does not affect the response to viral infection.

## DISCUSSION

TLR agonists have been used as important mucosal adjuvants in vaccine development; however, their roles in immune tolerance and activation of innate and adaptive immunity are still incompletely characterized. In this study, the immune response to intranasally administered TLR agonists was determined in TLR agonist-exposed SPF mice. We found that following exposure to matching TLR agonists, inflammatory cytokine responses to challenge with agonists of TLR2 or TLR4 were desensitized in URT mucosa; however, the responses to agonists of TLR3 or TLR9 were maintained. Moreover, the B cell response to agonist-adjuvanted antigens was substantially attenuated in mice exposed to extracellular TLR agonists. In contrast, the responses of both B and T cells to intracellular TLR agonists were vigorously induced. The findings in this study are summarized in Table 2 and demonstrate that the innate and adaptive immune responses are shaped differently by extracellular and intracellular TLRs in URT and related to TLR expression in immune cells.

**TABLE 2:** Summary of TLR-mediated URT mucosal immune responses following TLR agonist exposure

Cellular localization of TLRs	Types of TLRs	Inflammatory Cytokines	TLR expression in innate immune cells	Antibody response/ TLR expression in B cells	T cell response/ TLR expression in T cells
Extra- cellular	TLR4	↓^*a*^	CD11c^+^ monocytes ↓	↓	NC^*c*^
	TLR2	↓	CD11c^+^ monocytes ↓	↓	↓
Intra- cellular	TLR9	NC	CD11c^+^ monocytes ↓ Macrophages ↑^*b*^ Neutrophils ↑	NC	NC
	TLR3	NC	CD11c^+^ monocytes ↑	NC	NC

a ↓, downregulated.

b ↑, upregulated.

c NC, no change compared with agonist-nontreated mice.

We found that LPS exposure attenuated the LPS-adjuvanted antibody response but not the T cell response, indicating that the T cell response is not affected by desensitized mucosal inflammatory response to LPS, and that therefore LPS can be a mucosal T cell adjuvant. LPS-mediated B cell tolerance has been used for the suppression of allergy, such as inhibition of IgE production in airway hyper-reactivity in asthma (22). However, the underlying mechanisms are not clear. Our findings suggest that LPS-mediated reduction of TLR4 expression in B cells is involved in the mechanisms underlying this suppression. However, the T cell response to LPS-adjuvanted antigen stimulation was maintained accompanied by elevated expression of TLR4 in T cells. These results suggest that suppression of autoimmune T cells may require interruption of TLR4 signaling in T cells, in accordance with previous reports that loss of TLR4 solely in CD4^+^ T cells abrogates disease symptoms in the experimental autoimmune encephalomyelitis (EAE) model (33). Exposure to Pam3CSK4 induced inflammation tolerance and attenuated the Pam3CSK4-adjuvanted antibody production. However, unlike LPS, this exposure also affected the Pam3CSK4-adjuvanted T cell response with reduced TLR2 expression in T cells. Thus, Pam3CSK4 desensitizes both B and T cell responses. These findings suggest that Pam3CSK4 could also be used for the suppression of autoimmune diseases, especially those mediated by autoimmune T cells.

Intranasal exposure to CpG did not desensitize the inflammatory response to CpG stimulation but promoted the adaptive immune response to CpG-adjuvanted antigens. This finding is consistent with many studies in SPF mice, including ours, that CpG-adjuvanted vaccines through the i.n. route substantially induce antibody and T cell responses (34–36). Since CpG 1018 is licensed as an adjuvant for injection in the Heplisav-B vaccine (37), our results demonstrate that CpG also has a good effect as a mucosal adjuvant. A similar pattern was also found for Poly (I: C), an RNA agonist of TLR3, suggesting that effective adjuvant activity in the URT mucosa is shared by intracellular TLRs. High levels of IL-6 are involved in cytokine storm and correlated with pulmonary inflammation and extensive lung damage, which occur in several RNA viral infections, including SARS-CoV-2 (38, 39). Of notice, IL-6 production was much higher in Poly (I: C)-treated mice compared to CpG-treated mice in response to restimulation of matching TLR agonist (Fig. 4B and Fig. 3A) and TLR3 expression in CD11c^+^ monocytes increased while that of TLR9 decreased. Because TLR3 and TLR9 are commonly activated by RNA and DNA viruses, including intracellular bacteria, respectively, these findings suggest that different mechanisms are employed by RNA and DNA viruses for the activation of CD11c^+^ monocytes. The intolerance of the inflammatory response induced by the intracellular TLRs might be related to the severe inflammation often seen in respiratory infections by RNA viruses (40) or involved in viral-induced nasal allergic exacerbations through TLR3 activation (41). Verifying the link between the high levels of IL-6 and TLR3 expression in CD11c^+^ monocytes would help find interventional targets for a balanced inflammatory response to RNA viral infection.

Type I IFN regulates inflammatory cytokine production and can be induced by activation of TLR4 (IFN-β) (42) and TLR9 (IFN-α) (43). The regulation varies depending on the type of cells, the regulated inflammatory cytokines, and the type of IFN-I (44–46). Type I IFN probably involves the differential response of TLRs. However, the change of IFN-α induced by CpG does not play a role in TLR9 mediated IL-6 and TNF-α, suggesting that more mechanisms other than type I IFN involve the inflammatory cytokine response in NALT tissue with mixed cell types.

Different from that in SPF mice, the respiratory mucosa of human adults is exposed to the external environment and desensitized to agonists carried by commensal bacteria encountered in the respiratory tract with increasing age. Our results indicate that mucosal response to TLR ligands in SPF mice is more like in human infants or young children, which is not tolerated to either extra- or intracellular TLR ligands. Whereas the response is attenuated in SPF mice that preexposed to extracellular (LPS and Pam3CSK4) but not to intracellular TLR ligands CpG and poly (I: C). The observations in the ligand preexposed mice may reflect the situation in human adults as evidenced in human tonsils. These findings indicate that mucosal immune responses to TLR adjuvants are different between neonates and adults and that evaluation of mucosal vaccines and adjuvants in laboratory mice may not fully reflect the efficacy in humans.

In this study, we demonstrate that the TLR-mediated immune response in the respiratory mucosal site is tolerant to the extracellular TLR agonists but not to intracellular TLR agonists through regulating the expression of TLRs. The results of this study suggest that inflammatory responses induced by different TLR agonist impact B and T cell responses differently, which provides a basis for the use of TLR agonists as mucosal adjuvants and enhances our understanding of immune responses to bacterial and viral infections.

## MATERIALS AND METHODS

### Ethics statement.

This study was performed in strict accordance with the recommendations of the Guide for the Care and Use of Laboratory Animals of the IMCAS (Institute of Microbiology, Chinese Academy of Sciences) Ethics Committee. The protocol was approved by the Committee on the Ethics of Animal Experiments of IMCAS (Permit Number: SQIMCAS2018026). Mice were bred under specific-pathogen-free conditions in the laboratory animal facility at IMCAS. All animal experiments were conducted under isoflurane anesthesia, and all efforts were made to minimize suffering. Palatine tonsil tissues were obtained from healthy individuals (14–18 years) undergoing routine tonsillectomy. The donors had enlarged tonsils and experienced multiple tonsillitis. Consent was obtained from each of the donors for using the tissue sample in the research. The use of tonsil tissues was approved by the Research Ethics Committee (2018-162) of Beijing Children’s Hospital.

### Bacterial strains and virus.

The serotype M1 strain (90–226) of GAS was obtained from the University of Minnesota maintained on sheep blood agar, and grown in Todd-Hewitt broth supplemented with 2% neopeptone (THB-Neo; BD Bioscience, San Jose, CA) at 37°C in a 5% CO_2_ atmosphere. The nontypeable Haemophilus influenzae (NTHi) strain Sc70040 was obtained from Doctor Z. J. Shao, Chinese Center for Disease Control and Prevention, Beijing, and grown on chocolate agar at 37°C with 5% CO_2_. PR8, a mouse-adapted H1N1 influenza A virus, was obtained from Dr. B. Gao (IMCAS), cultured in the allantoic cavities of 9-d-old SPF embryonated hen eggs, and then incubated for 2 days at 35°C. The allantoic fluid was collected and stored at −80°C. The viruses were quantified in Madin Darby Canine Kidney (MDCK) cells and expressed as 50% tissue culture infective dose (TCID_50_).

### Mice, pretreatment with TLR agonists, immunization, and infection.

Female C57BL/6 (B6) mice (6–8 wk old) were purchased from Vital River Laboratory Animal Technology, the breeding colonies were all introduced by the Charles River Laboratories. SPF mice were pretreated with LPS intranasally to simulate the environmental exposure of human tonsils. For TLR agonist pretreatment, mice were anesthetized with an isoflurane/oxygen mixture for 1 min and inoculated intranasally through the nostrils dropwise with LPS (Beyotime, Haimen, China) (15 μg), Pam3CSK4 (Invivogen, San Diego, CA) (15 μg), CpG (Generay Co., Ltd. Shanghai, China) (ODN 1826/sequence 5′-TCCATGACGTTCCTGACGT-3′) (15 μg), or Poly (I: C) (Sigma-Aldrich, Brøndby, Denmark) (15 μg) in 10 μL PBS (5 μL of the suspension was delivered to each nostril) on five consecutive days. The 5-day treatment was selected based upon preexperiments that treatment more than 5 days did not further attenuate the cytokine responses. Control mice were given an equal volume of PBS. For stimulation, mice were inoculated intranasally with 30 μg of matching TLR agonist 48 h after the pretreatment. The doses used for each of the agonists were a minimum dose inducing the highest level of the inflammatory cytokine responses that were determined by preexperiments. For immunization, mice were inoculated intranasally with OVA (10 μg) combined with LPS (0.3 μg), CpG (10 μg), Pam3CSK4 (10 μg) or Poly (I: C) (10 μg) in 10 μL PBS (5 μL per nostril), and control mice were given OVA alone. The dose of antigen and TLR ligands was previously optimized. In the infection experiment, mice were inoculated intranasally with 1 × 10^7^ CFU of NTHi or GAS or 50 TCID_50_ of PR8 48 h after the pretreatment, and euthanasia 6 h, 12 h, and 72 h postinfection according to the preliminary data.

### Preparation of tonsil cells.

Tonsil tissues that were not required for pathological analysis were obtained within 3 h of surgery. The tissues were cut into small pieces with sterile scissors and digested in Hanks’ Balanced Salt Solution (HBSS) containing 1 mg/mL collagenase type I (Sigma-Aldrich, Brøndby, Denmark) for 40 min at 37°C. Digested tissue was subjected to filtration through a sieve with a sterile plunger to obtain single-cell suspensions, and the cells were washed in HBSS (4°C, 500 × *g*, 5 min). Red blood cell (RBC) lysis buffer was used to lyse RBCs in the cell suspension; lysis was then stopped by washing twice in fresh complete 1640 medium (4°C, 500 × *g*, 5 min). Tonsil cells were counted using a hemocytometer.

### Enzyme-linked immunosorbent assay (ELISA).

Mouse TNF-α, IL-6, IL-1β, and IL-10; and human TNF-α were analyzed for content using the Ready-SET-Go! ELISA kit (eBioscience, San Diego, CA, USA) according to the manufacturer’s instruction. Mouse IFN-α was analyzed with the Mouse IFN-alpha bioluminescent ELISA kit 2.0 (InvivoGen, San Diego, CA) as described by the manufacturer. OVA-specific serum antibodies were measured by endpoint ELISA as previously described (47). Corning Costar 96-well plates (Fisher Scientific, Waltham, MA, USA) were coated with OVA. A total volume of 100 μL samples were added to the plate and incubated at 37°C for 2 h. HRP-conjugated goat anti-mouse IgG (Southern Biotech, Birmingham, AL, USA) were used as the secondary antibody (1:4000). The reaction was developed by addition of TMB (Tiangen Biotech, Beijing, China). An ELx800 plate reader (BioTek, VT, USA) was used to measure absorbance at 450 nm, and absorbance at 570 nm was used as the internal control. The standard curve was generated by adding double diluted purified mouse IgG (Alpha Diagnostic Int. Inc, San Antonio, TX, USA) to anti-mouse IgG-coated wells. The concentration of immunoglobin levels was calculated according to the standard curve.

### Cellular staining and flow cytometry analysis.

Single-cell suspensions of NALT cells were stained for 30 min at 4°C with appropriate combinations of fluorochrome-conjugated maternal antibodies in the presence of 0.2% bovine serum albumin (BSA) and fixed in 4% paraformaldehyde in PBS. Cells were stained for surface markers with anti-CD284 (TLR4)-PE-Cyanine7 (SA15-21, Biolegend) or anti-CD284 (TLR4)-PE (SA15-21, Biolegend), anti-CD11c-PE (N418, eBioscience), or anti-CD11c-PerCP-Cyanine5.5 (N418, eBioscience), anti-CD19-APC (6D5, Biolegend), anti-CD3-FITC (17A2, eBioscience), anti-CD11b-PE-Cyanine7 (M1/70, eBioscience), anti-Ly-6G/Ly-6C (Gr-1)-APC (RB6-8C5, Biolegend), anti-F4/80-PE (BM8, eBioscience), and anti-CD282 (TLR2)-PE (CB225, Biolegend) as needed. For intracellular staining, fixed cells were permeabilized and stained in saponin (0.1% in PBS; Sigma) with anti-CD289 (TLR9)-FITC (M9.D6, eBioscience) and anti-CD283 (TLR3)-PE (11F8, Biolegend) in the presence of 1.5% BSA. Samples were analyzed using a FACSCanto flow cytometer (BD Biosciences) and FlowJo software (Treestar, Ashland, OR).

### Western blot.

A single cell suspension of NALT cells was centrifuged (4°C, 500 × *g*, 5 min). The cells were lysed on ice for 30 min with RIPA Lysis Buffer (Applygen, Beijing, China) containing protease inhibitors and phosphatase inhibitors (Applygen, Beijing, China); then, the mixture was centrifuged (4°C, 12,000 × *g*, 20 min). The supernatant was extracted and protein quantification performed using the bicinchoninic acid (BCA) kit (Tiangen Biotech, Beijing, China). SDS-PAGE electrophoresis was performed, then transferred to the polyvinylidene fluoride (PVDF) membrane (Millipore, Billerica, MA, USA). PVDF membranes were pre-blotted with 5% BSA (NOVON, Beijing, China) and incubated with the following antibodies overnight: anti-Phospho-NF-κB p65 (Ser536) (CST, Danvers, MA, USA), anti-NF-κB p65 (CST, Danvers, MA, USA) and anti-β-actin (Transgen Biotech, Beijing, China). The next day, HRP secondary antibody was used for labeling. The PVDF membrane was imaged using the enhanced chemiluminescence (ECL) (Yeasen, Shanghai, China) method with Tanon 5200 (Tanon, Shanghai, China). Protein expression was calculated using Image J software (NIH, New York, USA). The result is expressed as the ratio of P-NF-κB p65/NF-κB p65.

### Enzyme-linked immunospot (ELISpot) assays.

The number of SCPA-specific antibody-secreting cells (ASCs) was determined by ELISpot assays (35). In brief, PVDF membranes (Millipore, Billerica, MA, USA) were pre-wet with 35% ethanol and coated with SCPA (20 μg/mL) at 4°C overnight. A total of 1 × 10^6^ tonsil cells were plated in duplicate and incubated for 5 h at 37°C. The plates were washed and incubated with 50 μL of 1 μg/mL of HRP-conjugated goat anti-IgA or IgG per well. To detect antigen-specific IL-17A and IFN-γ-secreting cells, mouse IL-17A, IFN-γ ELISpot BASIC (HRP; Mabtech Mariemont, OH, USA) and human IL-17A Ready-SET-Go! ELISpot kit were used according to the manufacturer’s instructions. Our preexperiments showed that immunization through the intranasal (i.n.) route induced T cell response both in NALTs and spleens. Because NALT cells died in large numbers during culture splenocytes were used for IL-17A and IFN-γ ELISpot assays. A total of 5 × 10^5^ splenocytes or tonsil cells per well were cocultured with OVA or SCPA for 24 h. PMA and ionomycin-stimulated cells were used as positive controls. All plates were developed using procedures established by AEC (BD Biosciences, USA). Spots were enumerated with an ImmunoSpot Analyzer (Cellular Technology Ltd., OH, USA).

### RNA extraction and RT-PCR.

Total RNA was extracted from NALTs with TRIzol reagent (Invitrogen, Carlsbad, CA), and reverse transcription was performed using High Capacity cDNA Reverse Transcription Kits (Applied Biosystems, Life Technologies, CA, USA), as recommended by the manufacturer. Transcript products were amplified with SYBR Premix *Ex Taq* II (TaKaRa, Dalian, China) on a CFX96 (Bio-Rad) using the specific primer sets. The relative expression levels were evaluated using the 2−ΔΔCt method, and results were normalized to *GAPDH* expression as the internal control. The following RT-PCR primers (from Invitrogen) were used for amplification of mouse genes: *IFN-α*, 5′-GGACTTTGGATTCCCGCAGGAGAAG -3′ and 5′-GCTGCATCAGACAGCCTTGCAGGTC -3′; *GAPDH*, 5′-CATGGCCTTCCGTGTTCCTA -3′ and 5′-GCGGCACGTCAGATCCA -3′.

### Statistical analysis.

Statistical analyses were performed using GraphPad Prism software version 7.0. Unpaired, a two-tailed Student's *t* test or Mann-Whitney test was used to analyze the statistical significance between the two groups. One-way ANOVA with Tukey’s multiple-comparison test or Kruskal-Wallis with Dunn’s multiple-comparison test was used to analyze the statistical significance among more than two groups. The method of statistical analysis was based on the Shapiro-Wilk normality test. Significance was reached at *P* < 0.05. Data were presented as means ± SEM.
