# Supplementary material for: Differential Effects of Toll-Like Receptor Signaling on the Activation of Immune Responses in the Upper Respiratory Tract
Source: Microbiol Spectr. 2022 Feb 23;10(1):e01144-21. doi: 10.1128/spectrum.01144-21 (PMC8865572; doi:10.1128/spectrum.01144-21)
Supplement: SUPPLEMENTAL FILE 1 — Supplemental material. Download SPECTRUM01144-21_Supp_1_seq12.pdf, PDF file, 0.4 MB [file spectrum01144-21_supp_1_seq12.pdf]

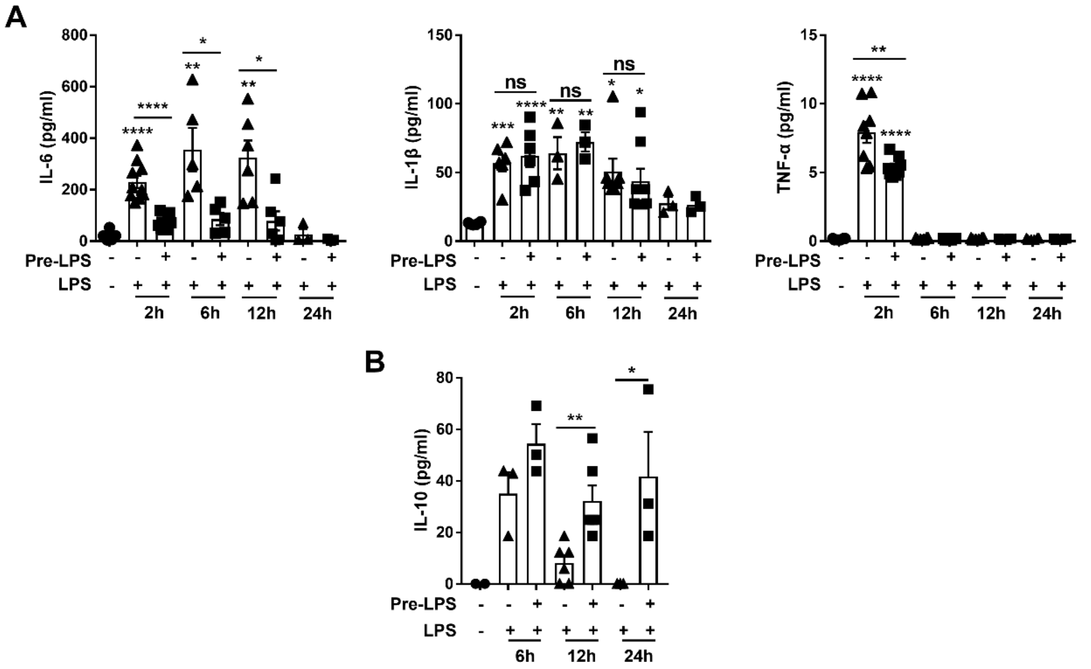

Fig. S1 Inflammatory cytokine assay at different time points. The experimental timeline is illustrated as in (Fig. 2). (A–B) The production of IL-6, IL-1β, TNF-α, and IL-10 was measured in NALT supernatant by ELISA at indicated times. Data are the mean ± SEM of 1–3 independent determinants (n=3–10). * $P < 0.05$, **$P < 0.01$, *** $P < 0.001$, **** $P < 0.0001$, ns, not significant. The asterisk above the bar indicates a significant difference compared to the first group.

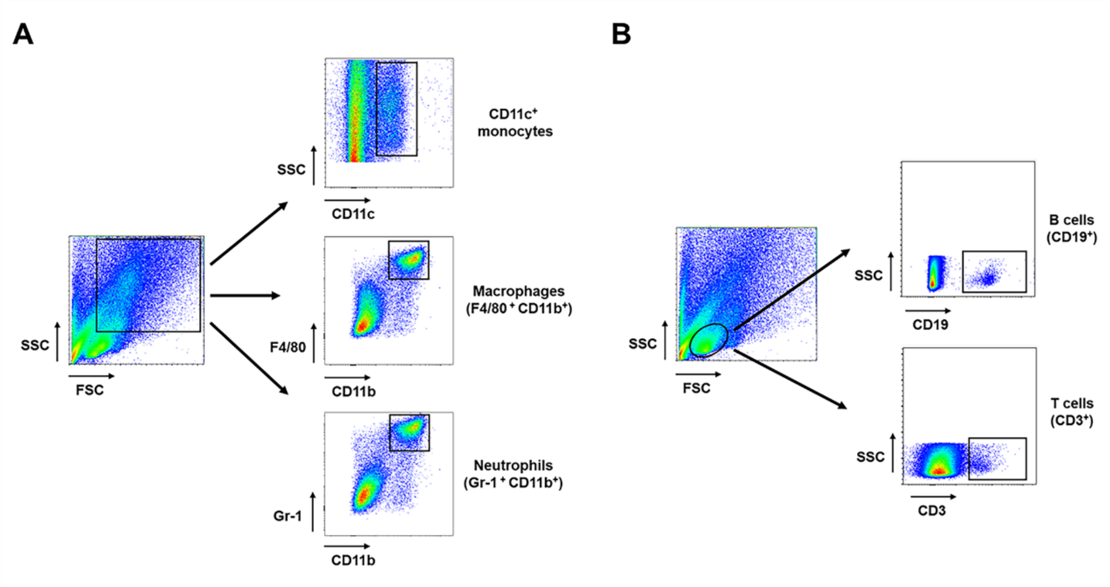

Fig. S2 Gating strategies for flow cytometric experiments. (A) Monocytes were gated by $CD11c^{+}$. Macrophages and Neutrophils were determined by F4/80 / CD11b and Gr-1 / CD11b, respectively. (B) B cells and T cells were gated by CD19 and CD3, respectively.

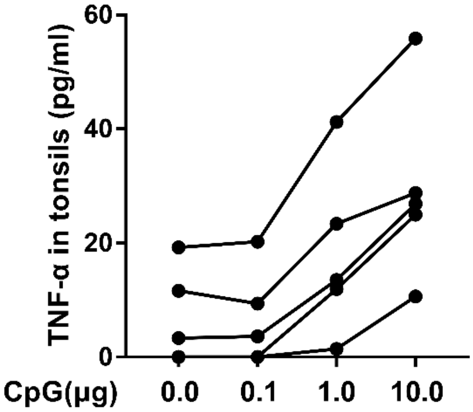

Fig. S3 CpG induced TNF-α production in human tonsil samples. Tonsil cells were stimulated with different doses of CpG, TNF-α production in culture supernatant was determined by ELISA after 24 hours. Each dot represents one donor (n=5).
